# Supplementary material for: Food Preference Assessed by the Newly Developed Nutrition-Based Japan Food Preference Questionnaire and Its Association with Dietary Intake in Abdominal-Obese Subjects
Source: Nutrients. 2024 Dec 9;16(23):4252. doi: 10.3390/nu16234252 (PMC11644194; doi:10.3390/nu16234252)
Supplement: Supplementary file 1 [file nutrients-16-04252-s001.zip › Nagai N et al. online_supplement/Nagai N et al. online_supplement.pdf]

ONLINE SUPPLEMENT

Table S1. Foods used to calculate the nutritional value of each item in the JFPQ.

Question items	Foods
1. Cakes (Western sweets)	Cheesecake unbaked, Cheesecake baked, Butter cake
2. Japanese sweets	"Kusa-mochi", "Sakura-mochi, Kansai style", "Mushi-manju"
3. Snacks	Potato chips regular, Potato chips fabricated, Corn snack extruded
4. Rice crackers	"Shoyu-senbei"
5. White rice	Nonglutinous rice well-milled
6. High-fat ramen noodles	The values were calculated from photo content.
7. Udon/soba noodles	The values were calculated from photo content.
8. High-fat Western style meal	The values were calculated from photo content.
9. Hamburgers	The values were calculated from photo content.
10. Fish	Sockeye salmon, Horse mackerel, Southern bluefin tuna
11. Vegetables	Head lettuce, Broccoli, Cherry tomato, Spinach, Burdock/root, Carrot/root
12. High-fat breads	Croissants, Fried bun with curry filling
13. Sweet breads	Baked bun with red bean pastes filling, Baked bun with strawberry jam filling
14. Deep-fried foods	"Karaage", "Tonkatsu", Tempura (The values were calculated from photo content.)
15. Meat	Chicken thigh meat with skin, Pork lean and fat, Beef lean and fat
16. Fruits	Navel oranges, Green flesh type kiwifruit, Pineapple, Grapes
17. Soft drinks (juice)	Valencia oranges reconstituted fruit juice, Cola
19. Non-sweet breads	White bread, French bread
20. Pastries	Yeast-leavened doughnuts, Apple pie
21. Soybean products	"Itohiki-natto", "Momen-tofu", "Kinugoshi-tofu"
22. Eggs	Hen egg, "Dashimaki-tamago"
23. Seaweeds	"Hijiki", "Wakame", "Mozuku"
24. Mushrooms	Winter mushrooms (Enokitake), "Shiitake", Beech mushrooms
28. Candy (drop, lollipop)	Sugar candy
30. Sweet chocolates	Milk chocolate, Sweet chocolate

The foods used to calculate the nutritional value of each item of the Japan Food Preference Questionnaire (JFPQ) are listed. An average value was calculated based on the nutritional value of these foods and used as the nutritional value for each item. For

some of the questions, the nutrient values were calculated from the foods shown in the photos.

Supplementary Figure Legend

Figure S1. Visualization of food preferences by each nutrient group in the non-obese and abdominal-obese groups via radar charts.

The visual analogue scale (VAS) scores of preferences for 25 food items of the Japan Food Preference Questionnaire (JFPQ) were summed for each of the four nutrient groups: carbohydrate, fat, protein, and dietary fiber (left). The carbohydrate and fat groups were further divided into sweet and non-sweet subgroups (right). The two radar charts illustrate differences in the strength and bias of nutrient preferences between the non-obese and abdominal-obese groups. The data are presented as a percentage of the total score relative to the maximum score. $^{*}p < 0.05$, $^{**}p < 0.01$, and $^{***}p < 0.001$ after adjustment for age and sex.
