# Supplementary figures and images for: Food Preference Assessed by the Newly Developed Nutrition-Based Japan Food Preference Questionnaire and Its Association with Dietary Intake in Abdominal-Obese Subjects
Source: Nutrients. 2024 Dec 9;16(23):4252. doi: 10.3390/nu16234252 (PMC11644194; doi:10.3390/nu16234252)

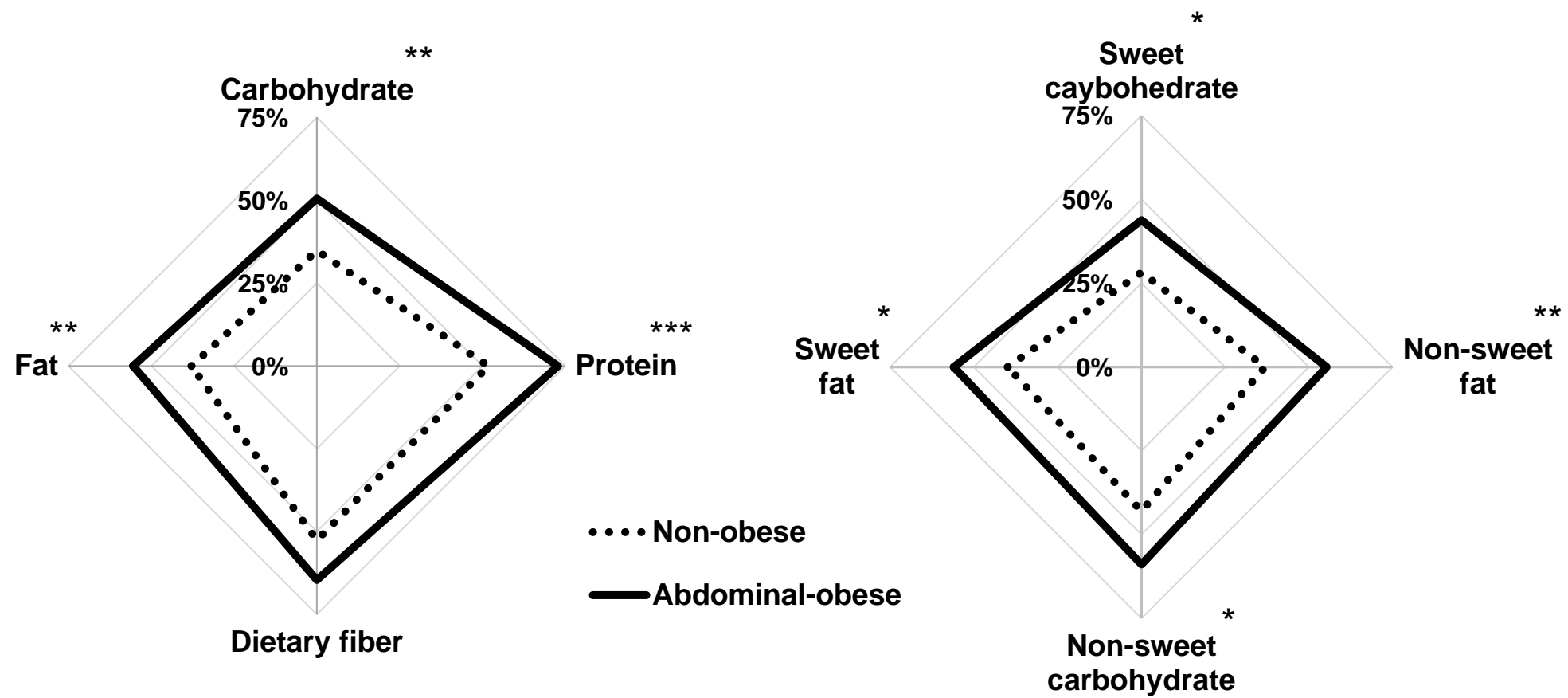

Figure S1

Supplement: Supplementary file 1 [file nutrients-16-04252-s001.zip › Nagai N et al. online_supplement/Figure S1.pdf]
